# Agathisflavone Inhibits Viability and Modulates the Expression of miR-125b, miR-155, IL-6, and Arginase in Glioblastoma Cells and Microglia/Macrophage Activation

**DOI:** 10.3390/molecules30010158

**Published:** 2025-01-03

**Authors:** Karina Costa da Silva, Irlã Santos Lima, Cleonice Creusa dos Santos, Carolina Kymie Vasques Nonaka, Bruno Solano de Freitas Souza, Jorge Mauricio David, Henning Ulrich, Ravena Pereira do Nascimento, Maria de Fátima Dias Costa, Balbino Lino dos Santos, Silvia Lima Costa

**Affiliations:** 1 Laboratory of Neurochemistry and Cellular Biology, Institute of Health Sciences, Federal University of Bahia, Av. Reitor Miguel Calmon S/N, Salvador 40231-300, BA, Brazil; enf.karinac@gmail.com (K.C.d.S.); irlalima@ufba.br (I.S.L.); cleonicemev@gmail.com (C.C.d.S.); ravenanascimento@ufba.br (R.P.d.N.); fatima@ufba.br (M.d.F.D.C.); 2 Center of Biotechnology and Cell Therapy, São Rafael Hospital, D’Or Institute for Research and Teaching, Salvador 41253-190, BA, Brazil; carolina.nonaka@hsr.com.br (C.K.V.N.); bruno.solano@idor.org.br (B.S.d.F.S.); 3 Institute Gonçalo Moniz, Fundação Oswaldo Cruz, Salvador 40296-710, BA, Brazil; 4 Department of General and Inorganic Chemistry, Institute of Chemistry, Federal University of Bahia, Salvador 40231-300, BA, Brazil; jmdavid@ufba.br; 5 Department of Biochemistry, Institute of Chemistry, University of São Paulo, Av. Prof. Lineu Prestes, 748-Butantã, São Paulo 05508-900, SP, Brazil; henning@iq.usp.br; 6 National Institute of Translational Neuroscience (INNT), Rio de Janeiro 21941-971, RJ, Brazil; 7 College of Nursing, Federal University of Vale do São Francisco, Av. José de Sá Maniçoba, S/N, Petrolina 56304-917, PE, Brazil

**Keywords:** polyphenolic compound, cancer, human miRNA, inflammatory mediators, microglia

## Abstract

Glioblastomas (GBM) are malignant tumours with poor prognosis. Treatment involves chemotherapy and/or radiotherapy; however, there is currently no standard treatment for recurrence, and prognosis remains unfavourable. Inflammatory mediators and microRNAs (miRNAs) influence the aggressiveness of GBM, being involved in the communication with the cells of the tumour parenchyma, including microglia/macrophages, and maintaining an immunosuppressive microenvironment. Hence, the modulation of miRNAs and inflammatory factors may improve GBM treatments. In this study, we investigated the effects of agathisflavone, a biflavonoid purified from *Cenostigma pyramidale* (Tul.), on the growth and migration of GBM cells, on the expression of inflammatory cytokines and microRNAs, as well on the response of microglia. Agathisflavone (5–30 μM) induced a dose- and time-dependent reduction in the viability of both human GL-15 and rat C6 cells, as determined by the MTT test, and reduced cell migration, as determined by cell scratch assay. RT-qPCR analysis revealed that agathisflavone (5 μM) down-regulated the expression of miR-125b and miR-155 in the secretome derived from GL-15 cells, which was associated with upregulation of the mRNA expression of IL-6 and arginase-1 immunoregulatory factors. Exposure of human microglia/macrophage to the secretome from GL-15 GMB cells modulated proliferation and morphology, effects that were modulated by agathisflavone treatment. These results demonstrate the effect of flavonoids on the growth of GBM cells, which impacts cells in the microenvironment and can be considered for preclinical studies for adjuvant treatments.

## 1. Introduction

Glioblastoma (GBM) is the most prevalent and aggressive primary brain tumor, exhibiting a high degree of invasiveness and resistance to treatment, with a dismal prognosis and an average life expectancy of approximately 14 months following diagnosis [1]. The World Health Organization (WHO) has classified GBM as a grade IV astrocytoma [2,3,4]. The incidence of GBM is estimated to be between 2 and 3 cases per 100,000 individuals, corresponding to approximately 14.9% of primary brain tumours and 51% of malignant gliomas. It represents approximately 2% of all types of cancers. The current treatment for GBM is a combination of surgical resection, radiotherapy, and chemotherapy. Among chemotherapeutic drugs, nitrosoureas (e.g., carmustine and lomustine) and alkylating agents, such as temozolomide (TMZ), stand out as particularly efficacious. Nevertheless, the therapeutic efficacy of this approach has been found to be limited due to the high recurrence rate, which is associated with the cellular adaptation and modulation of the surrounding microenvironment observed in GBM). Additionally, studies have indicated that stem cells present in the glioma microenvironment may contribute to tumour resistance against therapeutic approaches involving radiochemotherapy [4,5,6,7].

Studies have demonstrated that glioma cells regulate the expression of cytokines and other inflammatory mediators produced by microglial cells, immune cells responsible for innate immunity in the central nervous system (CNS), and stimulate the secretion of growth factors such as transforming growth factor β (TGF-β), which contributes to tumour proliferation [6,8,9,10]. Most recently, microRNAs (miRNAs), non-coding molecules, and post-transcriptional silencers have been identified in GBM. These molecules can be found in the cytosol or packaged in extracellular vesicles designated as exosomes [11,12], and they appear to be involved in tumour maintenance. Among the miRNAs that have been the subject of the most extensive study in GBM, miR-124, miR-146a, miR-155, and miR-21 have been identified as key regulators of GBM progression. miR-125 has been shown to play a crucial role in oncogenic upregulation and is associated with the STAT3 signalling pathway, contributing to the proliferation, migration, and invasion of the tumour cells. Furthermore, it has been associated with the growth and differentiation of cancer stem cells (CSCs) and cellular resistance to radiotherapy and chemotherapy. Another miRNA involved in glioma proliferation is miR-155. Studies have shown its potential in promoting glioma cell proliferation by downregulating Max interactor-1 (MXI1), a c-Myc promoter antagonist involved in transcription activation and promoting cell proliferation [11,12,13,14,15,16]. However, the precise role of these miRNAs in GBM remains to be elucidated, and their regulatory mechanisms and involvement in terms of cell proliferation and degree of microglial activation in the GBM microenvironment have yet to be defined [15,16]. In this context, there is a growing interest in the investigation of therapeutic alternatives for this type of tumour and in the identification of new targets that allow for the development of more effective therapeutic strategies.

Studies have demonstrated the antitumor potential of flavonoids. These molecules are natural phenolic compounds originating from the secondary metabolites of plants. They are known for their beneficial effects on health, involving antioxidant, anti-inflammatory, antiviral, and even anticancer properties [17,18,19,20,21,22,23]. Agathisflavone is a biflavonoid product of the conjunction of two molecules of apigenin. Its effects on CNS cells have been characterised, with emphasis on the neuroprotective and immunomodulatory effects on microglia [18,19]. Recently, we demonstrated that agathisflavone is selectively toxic for GBM cells of the GL-15 and U-373 lineages and is capable of reducing cell migration and inducing the differentiation of these cells towards a neural progenitor phenotype expressing astrocytic markers and neuronal events associated with expression-reduced constitutive and phosphorylated STAT3 (pSTAT3) [24]. On the other hand, we also demonstrated that agathisflavone can act directly on human and murine microglia, modulating inflammatory phenotypes [19,20,21,22,23,24,25,26]. In this context, the present study investigated whether the flavonoid agathisflavone is capable of modulating the expression of components of the inflammatory response and onco and inflammo miRNAs and the association between these effects and the viability of GBM cells as well as the impact on microglia state of activation.

## 2. Results

### 2.1. Agathisflavone Induces Toxicity and Inhibits Migration of Glioma Cells

**Figure 1:**
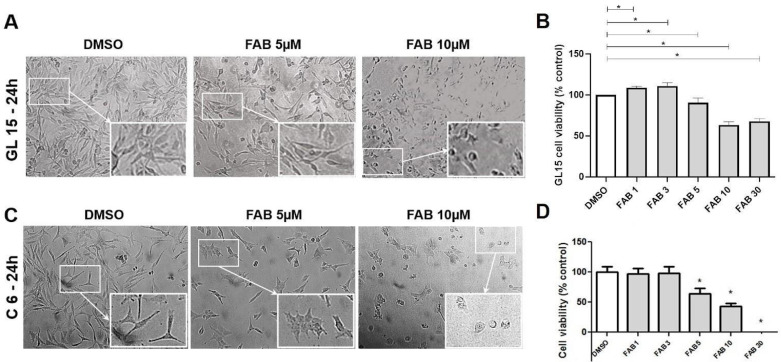
Cytotoxicity of agathisflavone to human GL15 and rat C6 rat glioma cells. The cells were treated with the flavonoid dilution vehicle (0.03% DMSO) or with agathisflavone (FAB) at a concentration of 1 to 30 µM, and the cell morphology and cytotoxicity was assessed after 24 h of exposure. (**A**,**C**) Phase contrast photomicrographs of GL15 and C6 cell cultures in control conditions or exposed to agathisflavone (5 or 10 μM) for 24 h; obj. ×20 scale bar = 100 μm. (**B**,**D**) Analysis of cell viability by the MTT assay in GL15 and C6 cell cultures exposed to agathisflavone at different concentrations; the results are expressed as the mean percentages ± SD (*n* = 3) in relation to the control group, which was considered 100%. (*) Statistically different, significance *p* < 0.05.

**Figure 2:**
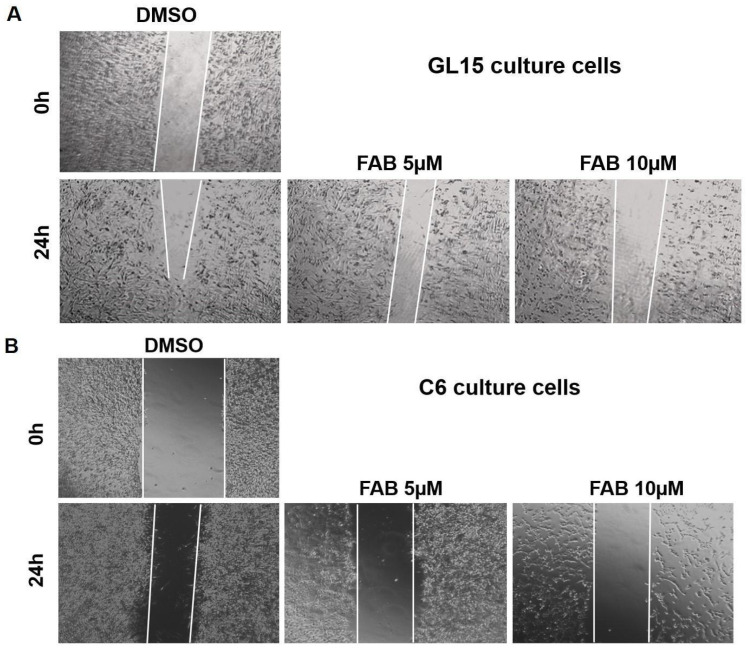
Effects of agathisflavone on migration of human GL15 (**A**) and rat C6 glioma (**B**) cells. The cell cultures were treated with the flavonoid (FAB) at a concentration of 5 and 10 µM or maintained under control conditions (0.01% DMSO). Migration was assessed after 24 h exposure by the Scratch assay; (obj. ×10).

First, we evaluated the effects of agathisflavone on cell viability in cultures of GL-15 human glioblastoma cells and C6 rat glioma cells. We observed that after 24 h of exposure of GL-15 and C6 cells to the flavonoid (1–30 µM), there was a dose-dependent reduction in cell viability, as measured by the MTT test, and in the cellularity from the concentration of 5 µM (Figure 1A–D). Furthermore, in the scratch test on cell monolayers, the cell-free area in cultures treated with 5 and 10 µM agathisflavone was preserved, indicating that the cells did not migrate (Figure 2A,B). On the other hand, in control cultures treated with DMSO (0.03%) cells that migrated to the scratched area after this period tending to close the cell monolayer.

### 2.2. Agathisflavone Modulates miRNAs and Cytokines Expression in GBM Cells

**Figure 3:**
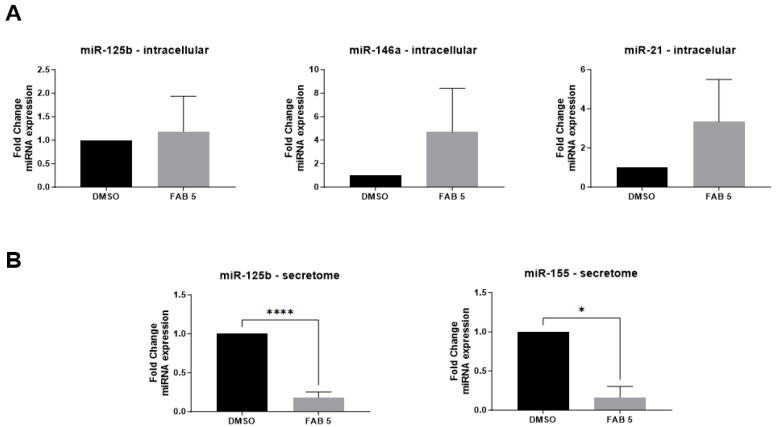
Effect of the flavonoid agathisflavone (FAB) on the regulation of miRNAs (miR) in intracellular and in the secretome of GL-15 human GBM cells by RT-qPCR. (**A**) intracellular expression of miR-125b, miR-146a and miR-21n; (**B**) extracellular expression of miR-125b and miR-155. Results were expressed as mean values ± SD (*n* = 3) and compared to control (0.005% DMSO) expression; FAB 5: agathisflavone at 5 µM. significance was determined by an unpaired *t*-test; (****) Statistically different, significance *p* < 0.0001; (*) Statistically different, significance *p* < 0.05.

Once the effects on viability and migration were determined, we evaluated the effect of agathisflavone on the regulation of expression of miR-146a, miR-125b, miR-21, and miR-155 miRNAs in human GL-15 GBM cells, exposing cells to an effective but subtoxic concentration of the flavonoid (5 µM) (Figure 3). Cellular miR-125b, miR-146a, and miR-21 expression levels were detected but were not significantly changed after agathisflavone treatment. On the other hand, agathisflavone significantly downregulated the expression of miR-125b and miR-155 in the secretome of GBM cells (Figure 3).

**Figure 4:**
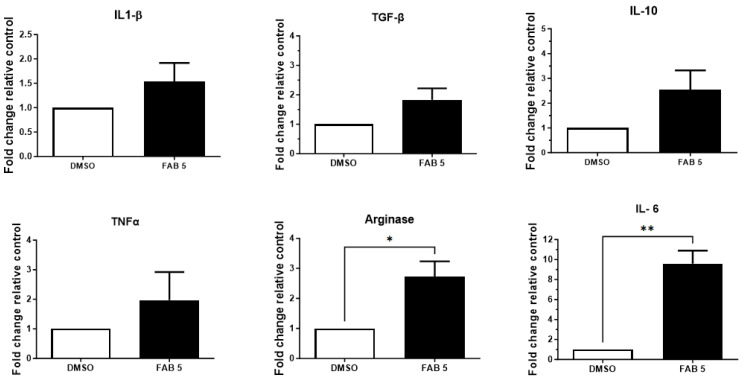
RT-qPCR analysis for mRNA cytokines’ expression in a culture of GL15 human glioblastoma cells. Expression of IL1-β, TGFβ, IL-10, TNFα, arginase 1 and IL-6. Results were expressed as means ± SD (*n* = 3) and compared to control (0.005% DMSO) expression; FAB 5: agathisflavone at 5 µM; (**) Statistically different, significance *p* < 0.005; (*) Statistically different, significance *p* < 0.05.

The effects of the flavonoid agathisflavone on the mRNA expression of mediators involved in inflammation were also investigated in human GL15 cells. We observed that agathisflavone tended to increase the expression of mRNA for mediators IL1-β, TNF, and TGFβ and significantly upregulated mRNA transcription coding for IL-6 and arginase-1 (Figure 4).

### 2.3. Treatment of GBM Cells with Agathisflavone Affects the Microglia State of Proliferation

**Figure 5:**
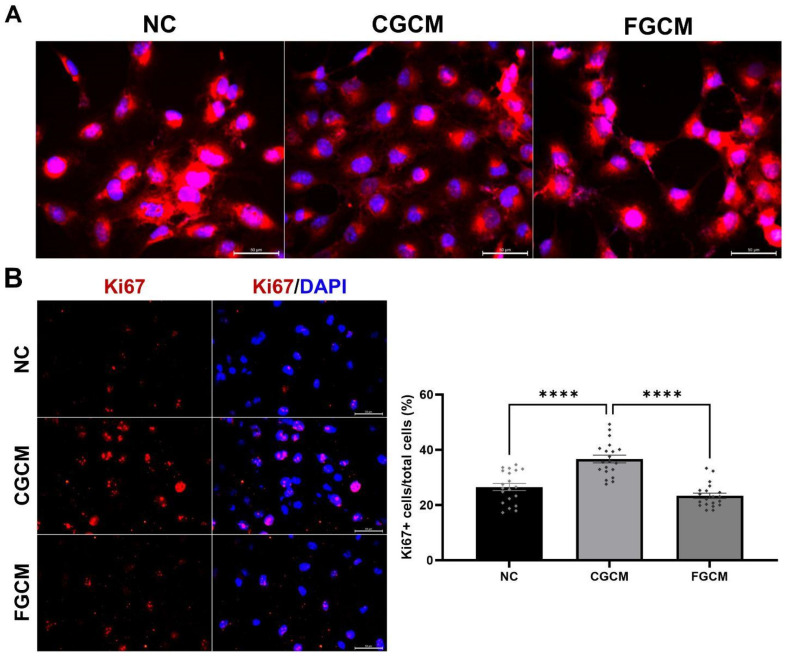
Effect of treatment with human GL15 GBM agathisflavone on the morphology and proliferation of human C20 microglia. GBM cells were treated for 24 h with agathisflavone (5 µM) or maintained in control condition (0.005% DMSO), and conditioned medium (CM) was collected after 24 h treatment. C20 microglia were exposed to fresh control medium (CM), to control CM of GBM cells (CGCM), or to agathisflavone GL15-treated CM (FGCM) for 24 h. (**A**) Cell morphology was assessed by phase contrast microscopy and proliferation by immunofluorescence for Ki67+ expression (red); the nuclear chromatin was stained with DAPI (blue); Obj. ×20, scale bar = 50 µM; the images are representative of three independent experiments. (**B**) Quantification of C20 microglia after 24 h of treatment in the different conditions; cells were counted in 20 aleatory fields in three independent cultures and were tested for significance using one-way ANOVA followed by Tukey’s post-hoc test; data presented as mean changes ± SEM times of controls. (****) Statistically different, significance *p* < 0.0001.

Considering the effects of agathisflavone on the expression of miRNA and inflammatory factors and the complexity of cell interaction in the tumour microenvironment, we investigated the effect of treatment with GBM cells on the phenotype and proliferation of microglia. For this, human C20 microglia was subjected to the conditioned medium (CM) from GL-15 cells treated or not for 24 h with the flavonoid (5 µM), and effects on cell morphology and proliferation, two features of the state of activation of microglia/macrophages, were analysed after 24 h of exposure (Figure 5A,B). We observed that C20 microglia in control conditions (fresh culture medium, CN) maintained their typical polygonal morphology, with about 36% of cells in a state of proliferation (Ki67^+^ cells). In C20 microglia cultures treated with the condition medium of GL15 GMB cells in control conditions (CGCM), the cell amoeboid morphology was predominant, and there was a significant increase in the proportion of proliferating cells (Ki67+). However, in microglia cultures treated with the CM of GBM cells treated with agathisflavone (5 µM) (FGCM), there was a significant reduction in the number of proliferating cells compared to cultures treated with control CM of GBM cells (CGCM), similar to that of negative control cultures, and cells assumed a branched phenotype.

## 3. Discussion

In this work, we explored the antitumor potential of the flavonoid agathisflavone, evaluating its effect on cytotoxicity, cell migration, and the regulation of tumour microenvironment mediators. For this, MTT and scratch assays were performed on both glioma cell lines, GL-15 and C6, in order to validate the effects and effective concentrations of these highly proliferative tumor cells. As shown in our results, in monocultures of glioma cells of the C6 and GL15 lineage treated directly with the flavonoid agathisflavone, concentrations equal to or greater than 5 µM were cytotoxic, with a significant reduction in cellularity. Here, we also showed in migration assays the capacity of agathisflavone to negatively regulate the migration of cells in the culture of C6 and GL15 glioma lineages. The inhibition of tumour proliferation is a property that has been described in the literature for several classes of flavonoids, and studies have shown the potential of flavonoids in regulating molecules associated with tumour cell migration, such as matrix metalloproteinases (MMPs), TGF-β, and vascular endothelial growth factor (VEGF), among others [20,21,22,23]. This finding is in accord with our previous study that demonstrated a selective effect of agathisflavona for U251 and U87 GBM cells [24]. Flavonoids such as isorhamnetin inhibited the cell proliferation of human cancer cell lines, negatively interfering with the cell cycle at the G2/M phase, in addition to inducing programmed cell death and autophagy in human cancer cells [25,26,27,28]. Moreover, we showed that the flavonoids 5-hydroxy-7,4′-dimethoxyflavone, casticin, apigenin, and penduletin, obtained from *Croton betulaster* leaves, and the glycosylated flavonoid rutin, obtained from *Dimorphandra mollis* pods, inhibited the proliferation of a human GBM cell line (GL15); additionally, rutin and casticin also negatively regulated the levels of VEGF and TGF-β1 [29], which involved increases in the migration of glioma cells [30,31,32].

In the present work, we also observed that agathisflavone was able to positively regulate the expression of the inflammatory cytokine IL-6 and the regulatory factor arginase, associated with increased response against the tumour. Here we also demonstrated that microglia treated with the conditioned medium from GBM cells entered into a state of activation, an effect that was regulated by exposing the tumour cells to the flavonoid. Some studies suggest that GBM cells modulate the expression of cytokines, chemokines, and growth factors present in the tumour microenvironment, where many of these mediators are produced by microglia [6,33,34]. Microglia present in the tumour microenvironment can suppress the immune response from the production of arginase 1 (Arg1) and pro-inflammatory and proliferative microglia have the potential to drive the progression of GBM [35,36]. Moreover, studies have shown that GBM cells inhibit the ability of microglia to produce pro-inflammatory cytokines such as TNF, IL-6, and IL1-β and stimulate the secretion of transforming growth factor β (TGF-β) and IL-10, a phenomenon correlated with increased expression of STAT-3 [6,9,10,37,38,39]. Previously, we demonstrated that reduction in cell viability of both human GL-15 and U373 GBM cells by agathisflavone was associated with modulation of STAT-3 signalling [24].

Agathisflavone also modulated the expression of miR-146a, miR-125b, and miR-21 in GL-15 human GBM cells and significantly downregulated the expression of miR-125b and miR-155 in the GL15 secretome. Expression of these miRNAs has been associated with the regulation of development, growth, and other cellular processes such as proliferation, differentiation, and metastasis [40,41,42,43,44]. MiR-146a inhibits the gliomagenesis process, suppressing the migration and proliferation of glioma cells, in addition to its ability to restrict the formation of glioma stem cells by regulating the Notch1 pathway, reducing proliferation, and inducing apoptosis. In addition, miR-146a also plays an important role in modulating the immune response, regulating the production of pro-inflammatory cytokines, and negatively regulating mediators of the inflammatory signalling pathway initiated by NF-kB in microglia [8,13,44,45]. Our results showed that miR-146a expressed in GBM was modulated by the flavonoid agathisflavone, which reinforces its anti-glioma potential. Studies show the role of miR-125b in up-regulating oncogenic activity and its association with the STAT3 signaling pathway, promoting proliferation, migration, and invasion of tumor cells. However, knowledge about the role of miR-125b in this scenario is still poor, and little is known about its mechanism of action in tumors. In work with HCT116 and HEK293 cells cancer cells, it was shown that miR-125b inhibition was associated with a reduction in cells’ growth and invasion. On the other hand, the use of miR-R125b mimics promoted the opposite effect [46]. Recently, our group identified that the flavonoid rutin promotes a significant reduction in the expression levels of miR-125b, both intracellular and extracellular, in GL-15 human GBM cells [47]. In this study, we also showed that agathisflavone downregulated the expression of miR-125b in GL-15 human GBM cells, which is in agree with an antitumor effect. Although the intracellular levels of this miRNA did not undergo significant changes, there was a significant reduction in the levels present in the secretome. These results indicate that agathisflavone acts as a regulator of miR-125b secretion, highlighting its potential role in modulating the tumor microenvironment. In another study with low-grade paediatric glioma, overexpression of miR-155 was associated with increased cell proliferation [48]. miR-155 is involved in glioma proliferation via downregulation of Max interactor-1 (MXI1) factor [11,12,13,14,15,16]. MXI1 is a c-Myc promoter antagonist involved in the transcription activation and promoting cell proliferation, and its downregulation contributes to cell proliferation and growth. In the work by Wu and Wang (2020) [42], the authors showed that in U87 GBM cells transfected with miR-155, there was a significant increase in the viability of tumour cells, as well as an increase in the rate of migration and cell invasiveness. On the other hand, transfection with an miR-155 inhibitor reduced this effect [14,42]. Studies have demonstrated that upregulation of miR-21 is associated with the pathogenesis of GBM, acting as an oncogene by promoting cell proliferation, invasion, and resistance to apoptosis. Masoudi et al. (2018) reported that miR-21 is a key factor in the pathogenesis of GBM, highlighting that overexpression of miR-21 results in a deregulation of crucial signalling pathways, such as PTEN/PI3K/AKT and TGF-β, contributing to tumour aggressiveness [49]. In this work, we demonstrated modulation of miR-21 expression after flavonoid treatment. This is relevant and may indicate that, although agathisflavone cannot directly reduce miR-21, it has the potential to modulate other crucial pathways.

In this study, we showed a downregulation of mi-R125b and miR-155 expression in the secretome originating from human GBM cells associated with a reduction in cell viability and migration, as well mRNA expression for ARG1 and IL-6 regulatory factors. In this sense, the downregulation of these miRNA in the secretome suggests a possible effect of the flavonoid on the regulation of cellular interactions and signaling in the tumor microenvironment. In fact, exposure of microglia to the CM from agathisflavone-treated GBM cells indicates changes in microglia phenotype and proliferation, which reinforces its potential as an antitumor molecule.

## 4. Materials and Methods

### 4.1. Glioma Cells Lines and Culture

The GL15 cell line was established from a human GBM [50] and was kindly donated by Marciene Tardy (Université Paris Este -Créteil Val de Marne). C6 glioma cells [51] were purchased from the Rio de Janeiro Cell Bank (BCRJ 0057) and were chosen for the present study considering their highly proliferation, migration, invasion, and resistance properties, similar to the GL15 cells. The glioma cells were cultured as described previously [52] until confluence in polystyrene plates (TPP, Trasadingen, Switzerland) in Dulbecco’s modified Eagle’s medium (DMEM; Cultilab, Campinas, Brazil) supplemented with 100 UI/mL penicillin G, 100 mg/mL streptomycin, 7 mmol/L glucose, 2 mmol/l L-glutamine, 0.011 g/L pyruvic acid, and 10% foetal calf serum (FCS). The immortalized primary human microglia C20 cell line was developed and characterized by Garcia–Mesa et al. (2017) [53] and was cultured in DMEM F12 50/50 medium as described by the authors. Cultures were maintained in a humidified atmosphere composed of 95% air and 5% CO_2_ at 37 °C. For experiments, cells were plated at a density of 5 × 10^4^ cells/cm^2^, and 24 h after plating, the medium was replaced with serum-free medium (SFM) for treatments.

### 4.2. Drugs and Treatments

The biflavonoid agathisflavone (FAB) was extracted from *Cenostigma pyramidale* (Tul.) E. Gagnon and G. P. Lewis (syn: *Poincianella pyramidalis*, *Caesalpinia pyramidalis*), as previously described by Mendes et al. [54], presenting 99% purity. It was dissolved in dimethyl sulfoxide (DMSO; Sigma Chemical Co, Saint Louis, MO, USA) at 100 mM, forming a stock solution, and was kept out of light at 4 °C until use. For treatments, the flavonoid was added directly into the fresh media at a final concentration or equivalent volume of DMSO and was analysed after 24 h. Control cultures were treated with DMSO (0.005 to 0.03%) considering the highest equivalent volume of the flavonoid stock solution adopted in each experiment. The highest concentration (0.03%) showed no significant effect on the parameters analysed compared to cells that did not receive the diluent.

### 4.3. Cell Viability Assay

The cytotoxicity of agathisflavone in cultures cells was determined using 3-(4,5-dimethylthiazol-2-yl)-2,5-diphenyltetrazolium bromide (MTT; Sigma). Confluent cells cultured in 96-well plates (TPP, Trasadingen, Switzerland) were exposed to agathisflavone (1–30 μM) or to the vehicle of dilution (DMSO 0.03%, control) for 24 h. Two hours before the end of the exposure time, the culture medium was replaced by a solution of MTT (5 mg/mL), diluted in DMEM, and incubated for 2 h in a humidified atmosphere with 5% CO_2_ at 37 °C. Thereafter, cells were lysed with 20% (*w*/*v*) sodium dodecyl sulphate (SDS), 50% (*v*/*v*) acetic acid, and 2.5% (*v*/*v*) 1 mol/L HCl. The plates were then kept overnight at 37 °C to allow the formazan crystals to dissolve. The absorbance at 540 nm was measured using a Varioskan Flash Spectrophotometer (Thermo, Waltham, MA, USA). Three independent experiments were conducted, with eight replicate wells in each condition.

### 4.4. Migration Assay

In order to assess whether agathisflavone affects the migration of C6 and GL-15 cells in isolated cultures or cell migration during glioma by interacting directly (co-cultures) with microglia, a scratch was created with a 200 µL pipette tip in the cultures. Cultures were rinsed once with DMEM to remove floating cells and were cultured with fresh serum-free medium containing DMSO (0.005%) or agathisflavone (5 µM or 10 µM).

### 4.5. RT-qPCR for Cytokines Expression

In order to assess mRNA expression, we removed the culture medium of GL15 cells and extracted total RNA using Trizol^®^ reagent (Invitrogen, Life Technologies, Carlsbad, CA, USA, 15596026) according to the manufacturer’s instructions. We then determined the RNA concentration and purity using the KASVI Nano Spectrum (cat# K23-0002). The RNA samples were treated with DNase using the Ambion (Singapore) DNA-free kit. cDNA was synthesised using SuperScript^®^ VILO™ MasterMix (Waltham, MA, USA). qPCR was conducted using TaqMan^®^ Gene Expression Assays (Applied Biosystems, Foster City, CA, USA), which contain specific TaqMan^®^ probes and TaqMan Universal Master Mix II with UNG (cat# 4440038, Invitrogen, Life Technologies™, Carlsbad, CA, USA). The assays corresponding to the genes quantified in this study were as follows: IL1β (Hs00580432_m1), TNFα (Hs00174128_m1), IL-6 (Hs00174131_m1), TGF-β (Hs00998133_m1), IL-10 (Hs00961622_m1), and arginase (Hs00163660_m1). We used the Quant Studio 7 Flex™ Real-Time PCR System (Applied Biosystems, CA, USA) to conduct real-time PCR. We followed the manufacturer’s instructions. ACTB and HPRT1 were used as reference genes to normalise gene expression data. The data were analysed using the 2−ΔΔCt method (Schmittgen and Livak, 2008) [55]. The results are the average of three independent experiments.

### 4.6. RT-qPCR for miRNA

Cellular miRNAs and the secretome (comprising soluble miRNAs and exosomes) were purified from GL-15 cells maintained in the control condition (0.005% DMSO) or treated with 5 µM agathisflavone for 24 h. The isolation of these samples was conducted in accordance with the previously described methodology [14]. To investigate the cellular miRNAs, approximately 1 × 10^6^ GL-15 cells were pelleted and mixed with 700 μL of QIAzol Lysis Reagent from the miRNeasy kit (Qiagen, Hilden, Germany). The isolation of miRNAs from the cell culture and supernatant was conducted using the miRNeasy Serum/Plasma Advanced kit (Qiagen). For the supernatant, a volume of QIAzol Lysis Reagent equal to five times that provided by the manufacturer was added. The samples were then vortexed for one minute. The recommended volume of chloroform was added to each kit, and the samples were vigorously mixed for 15 s. They were then incubated at room temperature for three minutes. Subsequently, the samples were subjected to centrifugation at 12,000× *g* for 15 min at 4 °C. The aqueous phase was then collected and transferred to a new 1.5-mL tube (approximately 350 μL). A volume of 1.5 times (525 μL) of 100% ethanol was then added and homogenised. Subsequently, the samples were transferred to the column (RNeasy MinElute spin column) provided by the manufacturer and were centrifuged for 15 s at a minimum of 10,000× *g* at room temperature. The liquid that passed through the column of each sample was discarded, and the column was washed with a volume of 700 μL of Buffer RWT and centrifuged for another 15 s at ≥10,000× *g*. Following the removal of the liquid that had passed through the column of each sample, the columns were washed with 500 μL of Buffer RPE and were centrifuged for 15 s at a minimum of 10,000× *g* at room temperature. Subsequently, the columns were washed with 500 μL of 80% ethanol and centrifuged for 2 min at a speed of ≥10,000× *g* at room temperature. Subsequently, the columns were transferred to newly labelled 1.5 mL tubes and were left with the cap open for five minutes in order to evaporate residual ethanol. A further 30 µL of RNase-free ultrapure water was added, after which the columns were centrifuged for one minute at maximum speed. The samples were subsequently stored at a temperature of −80 °C until the subsequent stage of the procedure was initiated. The miRNeasy Serum/Plasma kit (Qiagen) was employed in accordance with the manufacturer’s instructions to extract miRNAs from the cell culture supernatant. The miScript II RT Kit (Qiagen) was employed for cDNA synthesis, with 10 ng of RNA quantified by Nanodrop™ 2000 spectrophotometer (Thermo Fisher Scientific, Waltham, MA, USA), in accordance with the manufacturer’s instructions. The samples were incubated for 60 min at 37 °C, were then heated to 95 °C for 5 min, and were finally placed on ice. A total volume of 10 µL was prepared by combining 5 µL of diluted cDNA (1:20), 5 µL of SYBR™ Green PCR Master Mix (Thermo Fisher Scientific), and 1 µL of the commercial primer set miRCURY LNA (Qiagen). The miRNAs under investigation were miR-146a (hsa-miR-146a-5p), miR-155 (hsa-miR-155), and miR-125b (hsa-miR-125), which have previously demonstrated a significant impact on the modulation of tumour cell growth and the inflammatory profile of microglia. The expression of U6 snRNA (hsa, mmu) miRCURY LNA was employed as an internal control. Data were analysed using the ΔΔCt method. All experiments were conducted in triplicate and represent at least least three independent experiments.

### 4.7. Microglia Treatments and Staining

In order to analyse the effect of flavonoid treatment of GBM cells on microglia response, C20 microglia were plated in 24-well plates at a density of 2.5 × 10^4^ cells/cm^2^ and were treated with conditioned medium from GBM GL15 cells treated with agathisflavone (5 µM) or in control conditions. To determine morphology, microglia were stained with Sulforhodamine B (SRB, Sigma—Aldrich, Saint Louis, MO, USA, 230162). After 24 h treatments, the medium was removed, and the cells were fixed in situ by gently adding 50 µL of cold 50% (*w*/*v*) trichloroacetic acid solution (final concentration, 10%) incubated for 60 min at 4 °C. The supernatant was discarded, and the wells were washed five times with Milli-Q ultrapure water and air-dried. After washing, 50 µL of 0.4% (*w*/*v*) SRB solution was added to each well and incubated for 30 min at room temperature. The unbound dye was recovered, and the residual dye was removed by washing five times with 1% acetic acid. After three washes with PBS, the nuclei were stained for 5 min with 4′,6-diamidino-2-phenylindole (DAPI; Invitrogen; Thermo Fisher Scientific). The experiments were performed in triplicate. Quantification was analysed using ImageJ software version 1.54f (Wayne Rasband, National Institutes of Health, Bethesda, MD, USA). Images were observed and photographed using a fluorescence microscope (Leica, Singapore, DFC7000).

In order to analyze C20 microglial cells proliferation, the conditioned medium was removed 24 h after treatments, and cultures were washed three times with PBS. Then, the cells were fixed with cold methanol for 10 min at −20 °C. The cultures were then washed three times with PBS followed by 0.3% Triton X-100 treatment at room temperature for 10 min. The cells were blocked with 5% PBS/BSA for 1 h. Subsequently, they were washed three times with PBS and exposed to the primary anti-Ki67 antibody (1:100, mouse, eBioscience, San Diego, CA, USA, Cat# 14-5698, RRID: AB_10853185) diluted in 1% PBS/BSA, and kept under gentle agitation for 3 h in a dark chamber. The cells were washed three times with PBS, and the secondary antibody Alexa Fluor 594 goat anti-mouse IgG (1:1000; A11005, Life Technologies, Carlsbad, CA, USA) was added. The incubation with the secondary antibody was maintained under gentle agitation for 1 h at room temperature and protected from light. Afterwards, the cells were washed three times with PBS and incubated with 5.0 µg/mL of 4′,6-diamidino-2-phenylindole (DAPI, Molecular Probes, Eugene, OR, USA) at room temperature for 5 min for nuclear staining. The cells were washed three times with PBS. Experiments were performed in triplicate. Quantification of staining was performed using ImageJ 1.54f software (Wayne Rasband, National Institute of Health). Images were observed and photographed using a fluorescence microscope (Leica, DFC7000).

### 4.8. Statistical Analysis

The data were subjected to statistical analysis using GraphPad Prism 10.1.2. The initial step was to ascertain whether the values exhibited a Gaussian distribution. One- or two-way analysis of variance (ANOVA) was employed to analyse the results, with Tukey or Bonferroni post-tests being used for normal samples and Kruskal–Wallis followed by Dunn’s multiple comparison tests for non-normal samples. Additionally, the paired t-test was employed to assess the statistical significance of differences between two variables. The confidence intervals were defined at a 95% confidence level, with *p* < 0.05 considered to be statistically significant. Fold changes were calculated by dividing the mean value of the experimental group by that of the control group. In all figures, error bars represent the standard error of the mean (SEM) of at least three independent experiments.

## 5. Conclusions

The ensemble of results presented here reinforces the antitumor effect of the flavonoid agathisflavone and highlights the regulatory effects modulating onco miRNAs and inflammatory mediators expressed by GBM cells that influence the microglia state of activation. Considering that modulation of immune cells from the tumour microenvironment constitutes an important strategy for the treatment of malignant brain tumours, agathisflavone may be considered as a potential adjuvant for GBM treatments; however, further studies are needed to better understand the mechanisms involved in the effects and regulation of GBM cells’ interactions with immune effector cells.

## Data Availability

Data are contained within the article.
